# Striatal dopamine neurotransmission is altered in age- and region-specific manner in a Parkinson’s disease transgenic mouse

**DOI:** 10.1038/s41598-023-49600-5

**Published:** 2024-01-02

**Authors:** Jose Medina-Luque, Patrick Piechocinski, Paul Feyen, Carmelo Sgobio, Jochen Herms

**Affiliations:** 1 German Centre for Neurodegenerative Diseases (DZNE), Munich, Germany; 2 Centre for Neuropathology and Prion Research, Ludwig-Maximilians University, Munich, Germany; 3 Munich Cluster of Systems Neurology (SyNergy), Munich, Germany

**Keywords:** Parkinson's disease, Basal ganglia

## Abstract

Dopamine (DA) plays a critical role in striatal motor control. The drop in DA level within the dorsal striatum is directly associated with the appearance of motor symptoms in Parkinson’s disease (PD). The progression of the disease and inherent disruption of the DA neurotransmission has been closely related to accumulation of the synaptic protein α-synuclein. However, it is still unclear how α-synuclein affects dopaminergic terminals in different areas of dorsal striatum. Here we demonstrate that the overexpression of human α-synuclein (h-α-syn) interferes with the striatal DA neurotransmission in an age‐dependent manner, preferentially in the dorsolateral striatum (DLS) of PDGF-h-α-syn mice. While 3-month-old mice showed an increase at the onset of h-α-syn accumulation in the DLS, 12-month-old mice revealed a decrease in electrically-evoked DA release. The enhanced DA release in 3-month-old mice coincided with better performance in a behavioural task. Notably, DA amplitude alterations were also accompanied by a delay in the DA clearance independently from the animal age. Structurally, dopamine transporter (DAT) was found to be redistributed in larger DAT-positive clumps only in the DLS of 3- and 12-month-old mice. Together, our data provide new insight into the vulnerability of DLS and suggest DAT-related dysfunctionalities from the very early stages of h-α-syn accumulation.

## Introduction

Parkinson’s disease (PD) is a motor disorder characterized by the progressive degeneration of the nigrostriatal dopaminergic (DAergic) pathway and the presence of α-synuclein aggregations in intracytoplasmatic inclusions called Lewy bodies^1,2^. The involvement of the nigrostriatal DAergic system in the pathology was first noted in post-mortem tissue of PD patients in which depigmentation of the midbrain region was largely detected in those subjects^3,4^. In general, patients with Parkinsonism features are characterized by the drastic loss of DAergic neurons in the substantia nigra pars compacta (SNc)^5^; as a result, dopamine (DA) content is reduced and leads to an imbalance in the DAergic neurotransmission in the dorsal striatum, which affects motor and learning functions^6^. Although DA neuronal decline is exponential, it seems to unevenly affect the population of neurons in the SNc. While the ventral lateral tier of the SNc has the strongest neuronal loss, the population of neurons in the medial-dorsal tier is more resilient^7^. Similarly to what was observed post-mortem in midbrain regions, a selective vulnerability is also reflected earlier in the integrity of the striatal DAergic terminals, where DAergic fibers are more severely decreased in the putamen compared to caudate regions^8,9^.

α-synuclein, the main component of Lewy bodies, has been closely associated with the disruption of the DAergic system and the progression of PD^10–13^. In particular, besides its ability to modulate DA neurotransmission and bind dopamine transporter (DAT)^14,15^, several genetic studies identified duplication and triplication of the α-synuclein sequence (SNCA) in genetic PD^16^. Those patients are characterized by earlier PD-like symptoms onset, more extensive α-synuclein deposition, and more severe motor symptoms^17^. Several studies indicate that the degeneration of the nigrostriatal pathway is more evident in certain dopaminergic populations in the midbrain, and therefore, in their DAergic projections to the striatum^18–20^. Yet, it remains less understood how the early outbreak of human-α-syn accumulation (h-α-syn) and prolonged exposure to its high levels may interfere with the DA neurotransmission in discrete areas of the dorsal striatum. Thus, the present study aims to assess how continuous and steady overexpression of h-α-syn protein may differentially affect the DA neurotransmission of the dorsomedial (DMS) and dorsolateral (DLS) striatum, especially in the early phases of the synucleinopathology.

## Results

### Overexpression of h-α-syn differentially alters electrically evoked-DA release in the DLS in an age-dependent manner

**Figure 1:**
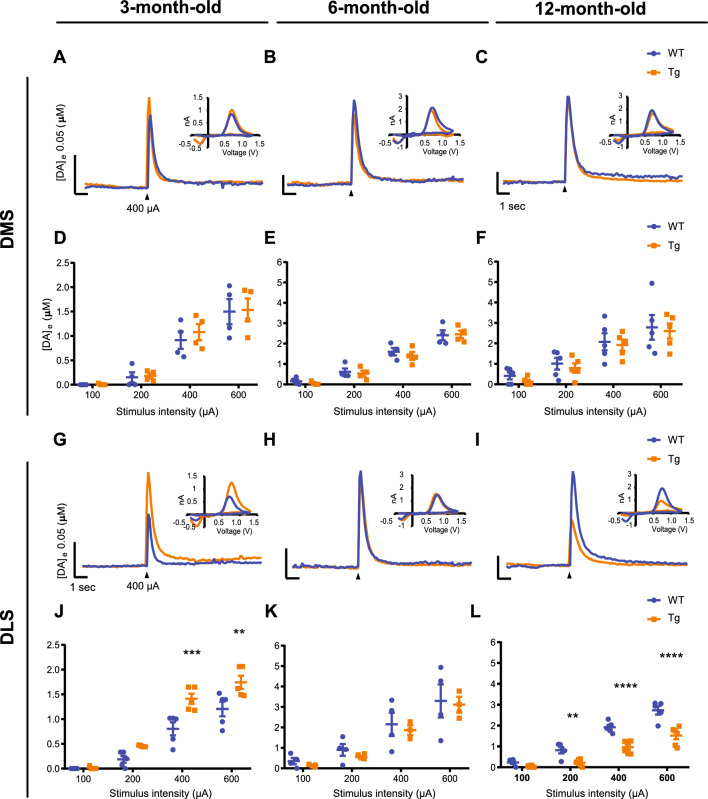
H-α-syn differently alters DA release depending on the age and striatal region. Representative traces and voltammograms of electrically evoked DA release at 400 µA in the DMS (**A**, **B**, and **C**) and DLS (**G**, **H**, and **I**) of Tg and WT mice. Input/Output curves of the average DA peak in the DMS (**D**, **E**, and **F**) and DLS (**J**, **K**, and **L**) of WT and Tg mice at the age of 3, 6, and 12 months by 100, 200, 400 and 600 µA electrical stimulus. Data represent mean ± SEM. (**p < 0.01; ***p < 0.001; ****p < 0.0001). n = 4 to 5 mice per group and 5 to 6 repetitions for striatum per region in each slice. Detailed statistics: Two-way ANOVA was used for statistical analysis, followed by Bonferroni’s *post hoc *tests. (**J)** F_(3,32) _= 4.109, Interaction Genotype x Stimulus: *p* = 0.0142; **p = 0.0014; ***p = 0.0003. (**L**) F_(3,36) _= 6.006, Interaction Genotype x Stimulus: *p* = 0.002; **p = 0.0075; ****p < 0.0001.

After electrically eliciting DA release neurotransmission with different stimulus intensities, the recordings displayed remarkable alterations in the dorsal striatum of Tg mice. While DA release appeared to follow similar dynamics in the DMS of both genotypes at all ages (Fig. 1A–C), those in the DLS were found to present striking alterations in Tg mice (Fig. 1G–I). Although both WT and Tg animals showed evoked DA release remained unchanged in the DMS (Fig. 1D–F), values in the DLS revealed an age-dependent alteration (Fig. 1J–L). In 3-month-old Tg animals, at an age where transgenic h-α-syn accumulation is supposed to start, DA levels revealed an increase in the DLS (Fig. 1J). In comparison with 3-month-old WT mice, the amplitude of DA release in Tg mice significantly increased by ~ 75% and ~ 44% in 400 and 600 µA intensity stimulations, respectively (Fig. 1J). Following similar experiments, DA release did not display any significant difference between Tg and non-transgenic mice at 6 months old age (Fig. 1K). Hence, the differences observed in the DLS of younger Tg mice faded at the age of 6 months. In further experiments in adult Tg animals (12-month-old), DA peak amplitudes were substantially lower in the DLS of Tg mice as compared to the same region in WT animals (Fig. 1L). Those significant reductions were observed with 200, 400, and 600 µA stimuli by ~ 75, 50, and 55%, respectively (Fig. 1L). As DAergic terminals exhibit particular firing patterns under physiological conditions, we further applied a burst-like stimulus (50Hz, 5p at 400 µA). The burst-like stimulus paradigm confirmed DA release data observed in the DMS and DLS of Tg mice at 3, 6, and 12 months of age (Supplementary Fig. 1). Taken together, our dataset revealed that the steady overexpression of h-α-syn preferentially affects DA release in the DLS.

### Striatal DA uptake exhibits slower kinetic in the DLS of Tg mice

**Figure 2:**
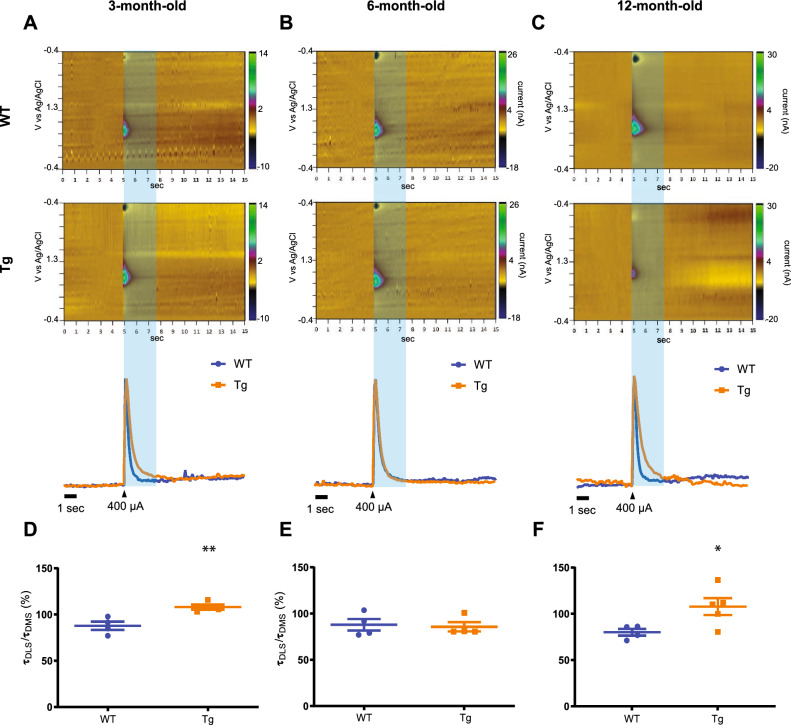
DA uptake is altered in the DLS in 3- and 12-month-old TG mice. Representative FSCV color plot graphs (top), normalized traces (bottom) for the DLS of WT (blue line) and Tg (orange line) mice at the age of 3 (**A**), 6 (**B**), and 12 months (**C**). Slope time constant ratio between DLS and DMS in WT and Tg at 3- (**D**), 6- (**E**), and 12-month-old (**F**) mice. Data represent mean ± SEM (*p < 0.05; **p < 0.01) from 4 to 6 repetitions of 4 to 5 WT and Tg mice. Detailed statistics: Unpaired two-tailed t Student´s was performed to compare the time constant ratio between WT and Tg. (**D**) 3-month-old mice: t_(6)_ = 3.853, **p = 0.0084. (**F**) 12-month-old mice: t_(7)_ = 2.567, *p = 0.0372.

Next, to examine whether the overexpression of h-α-syn may also interfere with the DA uptake, decay time (τ) was calculated from the slopes of 400 µA I/O stimuli between 10 and 50 ms after the highest amplitude value. (Fig. 2A–C, blue block). As DA release remained unchanged in the DMS at the different time points, the τDMS was considered as an internal control reference for the τDLS values. Kinetic analysis of the τDLS/τDMS ratio at 3 months of age showed a significantly increased of ~ 28% as compared to WT mice (Fig. 2D), reflecting DA level in the extracellular milieu persisted longer until it reaches baseline values. In contrast, no changes were found in 6-month-old Tg mice (Fig. 2E). Notably, the decay ratio in 12 months old animals displayed a significant increase of ~ 28% (Fig. 2F), showing similar as observed in 3 months old mice. Thus, these results indicate that the overexpression of h-α-syn also affects DA uptake in the DLS, specifically in 3- and 12-month-old mice.

### H-α-syn overexpression affects extracellular DA clearance in the DLS in both 3- and 12-month-old mice

During DA release and uptake, the number of DAergic neurons in the midbrain and the protein DAT plays a crucial role in the maintenance, clearance and recovery of DA^21,22^. As loss of DA-like neurons may be a direct cause of DA release and uptake disruptions observed at the age of 12 months of age, midbrain density neurons were calculated in the VTA and SNc. Similar to previous studies^23,24^, density values showed that the number of VTA and SNc dopaminergic neurons remain unchanged (Supplementary Fig. 2A,B). Next, DAT uptake functionality was assessed in the DAergic terminals by incubating the brain slices with cocaine (Coc, 3 µM, Supplementary Fig. 2) based on data obtained from trace studies in Tg mice at 3 and 12 months of age. In line with the previous results, extracting color plot graphs and traces corroborated baseline levels as well as τ ratio from I/O trials in 3- and 12-month-old Tg mice (Supplementary Fig. 2C,D). In 3-month-old mice, 15 min exposure to Coc, only showed differences in the τDLS/τDMS ratio between the genotypes but no changes in the DAT inward functions (Supplementary Fig. 2C,E). However, in 12-month-old mice, the ratio increased faster in Tg compared to WT littermates (Supplementary Fig. 2D,F). The dynamics displayed in both experiments may suggest that DAT functionality may probably be involved in mediating the persistence of DA in the extracellular space during neurotransmission in 12-month-old mice, but not in 3-month-old TG, where uptake alterations may be ascribed to a problem in DAT availability and membrane translocation.

### Enhanced and normal DA release are associated with motor learning performance in 3- and 6-month-old Tg mice

**Figure 3:**
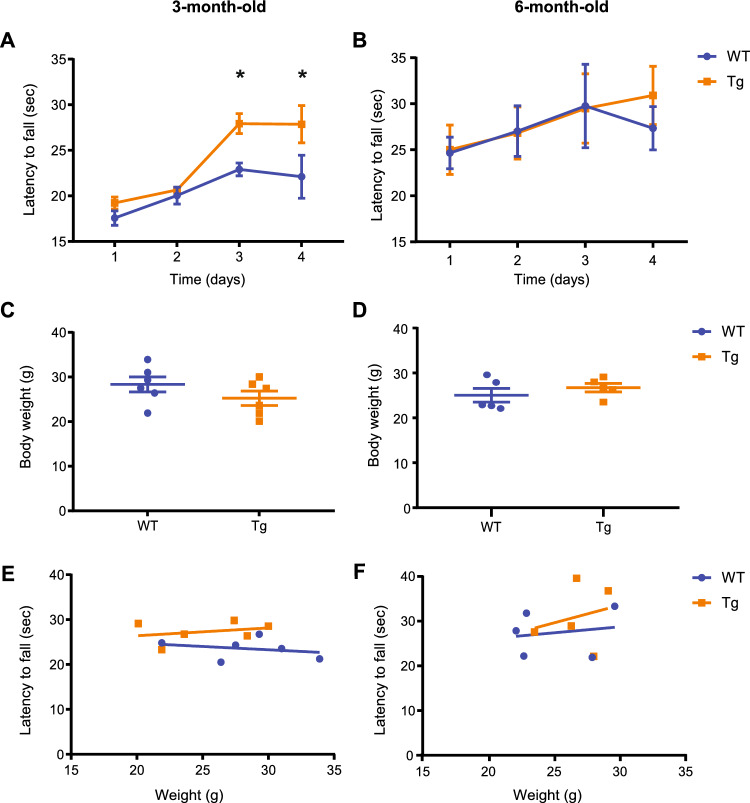
Procedural motor learning is enhanced at the age of 3 months old but remained the same at 6 months old in Tg mice. Graph depicting the latency to fall (sec) during the four days in which time on the rotor was measured at 3- (**A**) and 6-month-old (**B**) Tg and WT littermates. Each time point represents the average latency to fall (sec) of 10 daily trials. Comparison of body weight between WT and Tg mice at the first testing day at 3 (**C**) and 6 months of age (**D**). Scatterplot between the time on the rotor vs body weight at 3- (**E**) and 6- (**F**) month-old mice. Data were represented as mean ± SEM. Bonferroni’s post hoc tests: *p < 0.05. n = 5–6 mice per group. Detailed statistics: Two-way ANOVA was used for statistical analysis, followed by Bonferroni’s *post hoc *tests. (**A**) Genotype x Day interaction: F_(3,40)_ = 2.886, *p* = 0.0474. Pearson correlation analysis. (**E**) WT, *p* = 0.6151, r = − 0.2627; Tg, *p* = 0.5793, r = 0.2885; (**F**) WT, *p* = 0.7780, r = 0.1753; Tg, *p* = 0.7037, r = 0.2349.

DA neurotransmission has a crucial role in the consolidation and acquisition of motor learning skills^25,26^. As Masliah and colleagues correlated the disruption of the DA system with a deficit in the motor learning skills in 12-month-old Tg mice^23^, we aimed to elucidate whether DA alterations may also induce changes in the motor learning performance at 3 and 6 months of age. At 3-month-old, both Tg and WT littermates similarly improved their motor learning performance within the first two testing days. However, latency to fall significantly increased during the third and fourth testing day in Tg mice. Compared to WT, 3-month-old Tg animals stayed longer on the rod by ~ 21% and ~ 26% on the third and fourth day, respectively (Fig. 3A). Interestingly, no significant differences were found between WT and Tg mice at the age of 6 months old (Fig. 3B) confirming, as previously observed in Fig. 1, that DA changes have an impact in the motor performance. To dismiss any influence of the body weight in our final readout, mice were weighed on the first testing day. The statistical comparison showed no weight differences between groups at 3- and 6-month-old (Fig. 3C,D). In addition, to exclude the influence of body weight on mice performance a Pearson correlation was carried out with no significant differences (Fig. 3E,F). Therefore, we can conclude that our results were not influenced by body weight.

### Reorganization of DAT-like distribution in the dorsal striatum of Tg mice

**Figure 4:**
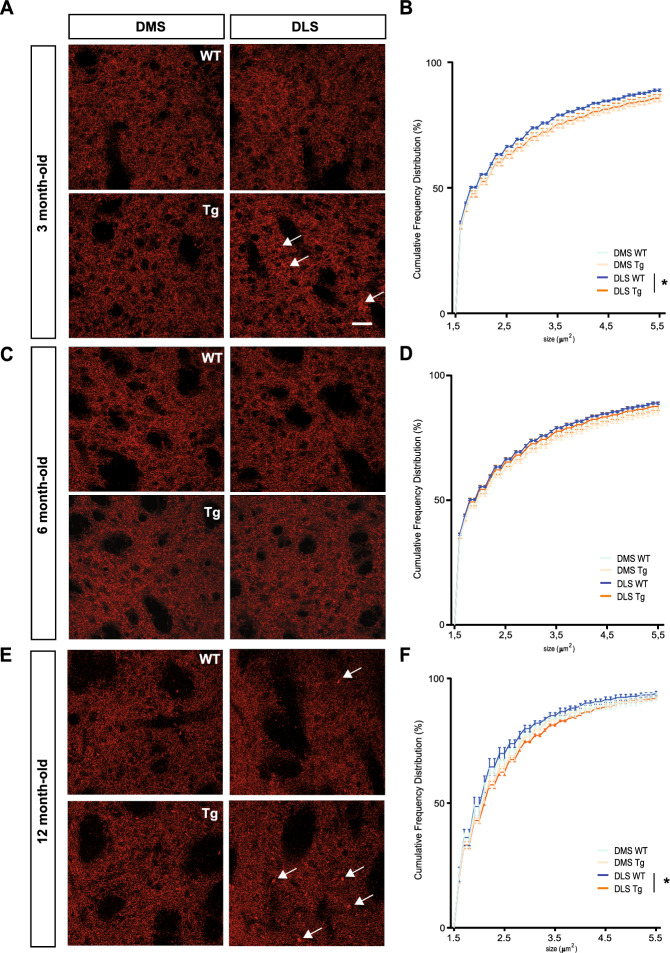
DAT-positive puncta analysis revealed an increased proportion of larger clumps in the DLS. Selected confocal microphotographies representing the staining for DAT in the DMS and DLS DAergic afferents of WT and Tg animals at the age of 3 (**A**), 6 (**C**), and 12 (**E**) months of age. White arrows highlight the aberrant aggregation or clumps of DAT staining. Images were acquired with an×40/1.4NA oil immersion objective. Graphs of Cumulative Frequency Distributions (CFD) in the DMS (WT, bright blue; Tg, bright orange) and DLS (WT, dark blue; Tg dark orange) at 3- (**B**), 6- (**D**), and 12- (**F**) month-old. Data were expressed as averaged CFD within the same genotypes (n = 4 animals per genotype), for each region. Bonferroni’s post hoc tests: *p < 0.05. Bars scale: 20 μm. Detailed statistics: Two-way ANOVA was used for statistical analysis, followed by Bonferroni’s *post hoc *tests. (**B**) Puncta Size main factor: F_(86,1044)_ = 1390, *p < 0.05. (**D**) Puncta Size x Region interaction: F_(255,688)_ = 0.4813, *p* > 0.9999. (**F**) Puncta Size main factor: F_(85,860)_ = 1173, *p < 0.05.

Since we previously observed alterations in the extracellular DA clearance during neurotransmission in the DLS of 3- and 12-month-old Tg mice, we performed fluorescence-based immunostainings to analyze DAT distribution in the dorsal striatum. While the DAT signal appeared to remain unchanged in the DMS and DLS in Tg mice compared to WT in terms of OD analysis and puncta density (Supplementary Fig. 3), the distribution pattern showed striking differences. Although we observed that DAT-like distribution was similar in the DMS of Tg and WT mice (Fig. 4A,C, and E, DMS), we found signs of accumulation of DAT immunolabelling in the DLS of the Tg mice (Fig. 4A,C, and E, DLS). These DAT-positive clumps appeared to be larger than the average size of DAT-like puncta and side-predominant since we found them mainly in the DLS of Tg mice (white arrows, Fig. 4). To confirm those observations, we carried out a deeper analysis of the diffuse DAT-like distribution by applying a threshold-based mask and displaying a CFD. In a comparison between both genotypes, DMS exhibited the same DAT-positive puncta size CFD between both genotypes at any age (Fig. 4B,D and F). However, in 3-month-old mice, the size distribution was shifted to the right in the DLS of Tg as compared to WT (Fig. 4B) indicating that the proportion of larger DAT-positive clumps was higher in Tg than in WT animals. In an equivalent comparison of 6-month-old mice, no differences were found (Fig. 4D). The comparison of 12-month-old mice yielded clear differences resembling those of 3-month-old, showing a higher proportion of larger DAT-like clumps in the DLS of Tg versus WT mice (Fig. 4F).

## Discussion

In the present study, we demonstrate the region- and age-dependent impact that h-α-syn overexpression has on striatal DA dynamics. Indeed, one of the most robust findings was the selective disruptions observed in DA release and uptake of the dorsal striatum of Tg mice. While DA neurotransmission in the DMS remained unaffected in Tg mice, DLS showed striking alterations across different mouse ages. FSCV ex vivo experiments revealed increased electrically evoked DA release in 3-month-old mice, and an impaired DA neurotransmission at 12 months (both with single and burst-like stimuli). Supporting our data, Lam and co-workers^27^ described a similar age-dependent effect, by performing in vivo microdialysis in the dorsal striatum of the Thy-αSyn transgenic mouse line (Line 61). Monitorization of extracellular DA content showed a strong decrease in the extracellular striatal DA levels in aged mice (14 months) but also a significant increase in DA content at an earlier age (6 months). We extended their investigation by using FSCV which has a better spatial resolution to discriminate DLS and DMS, and a much higher temporal resolution, to detect DA release (sub-second scale). The different onset of the neurotransmission-related deficit between Thy-αSyn and our PDGF-β Tg mice may be due to different h-α-syn expression levels. While PDGF-β Tg mice express moderate levels of h-α-syn (1.5-fold), Thy-αSyn mice expression is much higher, equivalent to tenfold of the physiological levels in human tissue^28^. Given that the PDGF-β promoter provides a steady transgenic expression of h-α-syn, the Tg model is particularly reliable for mimicking the pathological condition in human patients.

Together with the increased DA release in 3-month-old mice, Tg mice also showed improved motor learning skills in the rotarod test after four days of training. That enhanced ability disappeared at 6 months, together with any anomalies in DA neurotransmission in DLS. At 12 months, Tg mice again showed altered DA release, this time in terms of decreased neurotransmission, which correlates with the described motor dysfunctions in the rotarod task at 12 months^23^. Motor memory formation depends on DA neurotransmission in the DLS, as this region is critically involved in motor learning and motor control^29–32^. In line with our results, Giraldo and colleagues^33^ also concluded that overexpression of h-α-syn improved the performance of M20 mice in several motor tasks. But why does the α-synuclein overexpression enhance DA release in young animals?

It is well-established that α-synuclein actively participates during the key processes of neurotransmitter release. Among many mechanisms, α-synuclein facilitates DA release during burst firing stimulation in substantia nigra neurons^34^ and is also able to modulate neurotransmission by interacting with DAT through its C-terminal tail^19,35^. Our data indicate that DA clearance kinetics in DLS was slower in 3- and 12- month-old Tg mice when compared with WT animals. Further pharmacological experiments blocking DAT activity confirmed that the slow kinetics was driven by changes in DAT functionality at early and late ages. Similarly, Lundblad et al.^36^ described an early reduction of the striatal DA uptake rate in rats stereological injected with a viral vector designed to induce h-α-syn overexpression in the SNc. In cell culture, overexpression of α-synuclein and human-DAT (hDAT) identified an attenuation of DAT activity when DA was infused into the medium, suggesting possible changes either in the function or availability of the protein^14,35,37^. Comparable to our results, it has been shown that initial high levels of α-synuclein resulted in decreased extracellular DA clearance rate^14^. In contrast, experiments using either human embryonic kidney cells (HEKs) expressing hDAT and mice overexpressing h-α-syn or Thy-αSyn supported the opposite idea, describing an increasing or no change in DAT uptake activity, respectively^19,27^. In those cases, discrepancies with our dataset may be caused by differences in the experimental models due to different levels of h-α-syn protein expression and/or sampling methods, since they do not discriminate between the DMS and DLS. Indeed, DAT expression and functions affect striatal DA uptake rates and release magnitude differently along a dorsolateral to ventromedial axis gradient^38^. Interpretations for the slow kinetics observed in DA uptake at early and late stages of α-synuclein overexpression are complex and they might require more investigations. At later stages, the burden of prolonged α-synuclein overexpression might reduce DA release by impairing TH activity or vesicle release; however, the reduced DAT activity would be upheld in an attempt of maintaining ideal levels of extracellular DA in aged, damaged brains. In the early stages, acute and sudden increase of h-α-syn levels might directly impact DAT mobilization at the membrane level, where less functional transporters would directly result in reduced DA clearance and consequent increased overall release.

To date, several studies have supported that α-synuclein regulates not only DAT function but also its distribution^15,39–41^. We also reported the consequences that the overexpression of h-α-syn has over the normal distribution of DAT in the dorsal striatum. Although the optical density of DAT in the dorsal striatum was not different between genotypes, DAT immunolabeling revealed a singular reorganization of the characteristic DAT puncta distribution, specifically in the DLS. In particular, the microphotography analyses showed that DMS had an apparent regular DAT distribution at all ages when compared with control littermates. Remarkably, DLS was found to have a visible change in the dot-like appearance with the occurrence of DAT-positive clumps of the size larger than 2 µm, mainly at 3- and 12-month-old, but not at 6 months of age. Further CFD analysis proved that the widespread DAT pattern was modified and that the proportion of larger size spots was higher in the DLS when compared with the same region in WT mice. Previous studies using classical immunohistochemical methods in human striatal tissue reported the co-occurrence of α-synuclein and DAT labelling in caudate-putamen of PD patients. More importantly, they observed a redistribution of DAT individually or the DAT/α-synuclein complexes and described an increased size of what they called “DAT/α-synuclein clumps”^42^. This evidence is supported by other lines of investigation that observed a similar increase in the size of these complexes in different PD models^42–44^. The regulation of DAT distribution is determinant to balance the cytoplasmic and extracellular DA concentrations, in both striatal terminals and midbrain neurons. The redistribution of DAT in larger-sized clumps may be caused by an augmented internalization of the transporter from the membrane. This disproportioned internalization may directly interfere with DAT availability at the membrane level, and probably play a key role in the early-onset pathophysiology.

Interestingly, no changes were observed in DA neurotransmission or DAT redistribution in 6-month-old Tg mice in our experiments. The enhanced DA neurotransmission and DAT reorganization in 3-month-old Tg mice may result in adaptive mechanisms to counteract the impact of excessive α-synuclein. This is particularly true for PD because the unique properties of the nigrostriatal pathway guarantee high redundancy in their anatomical connections and activity^45^. For instance, DAergic axon arborization is not only dense in presynaptic boutons, but also exceptionally large. Thus, a single DA neuron can reach a large population of postsynaptic medium spiny neurons^46,47^. The redundancy of the “boutons reserve” in the DAergic innervation is also confirmed by the existence of functionally “silent” DAergic terminals^48^; thus a heterogeneity of individual DA boutons that can diversify their activity in response to insults is likely. Several adaptive mechanisms are surely in place at the very early stage of PD to preserve normal striatal neurotransmission and eventually recover from different sources of insults.

Taken together, the present work expands our knowledge of how changes in α-synuclein expression may interfere with striatal DAergic neurotransmission differently with age. At the early stages, acute and sudden increases of h-α-syn levels might directly impact DAT mobilization at the membrane level, where less functional transporters would directly result in reduced DA clearance and consequent increased overall release. At later stages, the burden of prolonged h-α-syn overexpression might reduce DA release by impairing DA homeostasis; however, the reduced DAT activity would be upheld in an attempt of maintaining ideal levels of extracellular DA in aged, damaged brains. Our data suggest that DLS may be more sensitive to the overexpression of α-synuclein because of DAT-related dysfunctionality and open a new promising perspective to address early adaptation in the progression of PD.

## Materials and methods

### Animals

Heterozygous transgenic mice overexpressing h-α-syn under the human platelet-derived growth factor β promotor (PDGF-β, Tg mice, Masliah^23^) were obtained from QPS Austria Neuropharmacology (Tg(PDGFB-SNCA)^4Ema^. Grambach, Austria). Wild-type (WT) littermates were considered as controls. Experiments were set at 3, 6, and 12 months of age. All animals were housed in groups under pathogen-free conditions, with food and water provided ad libitum (21 ± 2 °C, at 12/12-h light/dark cycle). All experiments were approved and performed by the Bavarian government (Az 55.2-1-54-2532-214-2016) and according to animal protection law. For each set of experiments described in this method section, independent cohorts of naïve mice were used**.** For this study, the experimental groups (WT and Tg mice) included both sexes. The reported study was carried out by following the recommendations within the ARRIVE guidelines.

### Fast scan cyclic voltammetry

#### Dopamine release and uptake

Electrically evoked DA release was recorded by using Fast-scan cyclic voltammetry (FSCV) in ex-vivo coronal slices containing dorsomedial (DMS) and dorsolateral (DLS) striatal regions. Briefly, isoflurane-anesthetized mice were euthanized by cervical dislocation, and the harvested brains were transferred into a semi-frozen cutting solution (Cutting solution: Sucrose 194 mM, NaCl 30 mM, NaHCO_3_ 26 mM, Glucose 10 mM, KCl 4.5 mM, NaH_2_PO_4_ 1.2 mM, MgCl_2_ 1 mM) and coronal slices of 250-μm thickness were sliced with a vibratome (Leica VT1000S. Wetzlar, Germany) pre-chilled at −4 °C. The coronal striatal slices were kept in an oxygenated artificial cerebrospinal fluid medium (α-CSF; NaCl 126, KCl 2.5, NaH_2_PO_4_ 1.2, CaCl_2_ 2.4, MgCl_2_ 1.2, NaHCO_3_ 25, glucose 11, HEPES 20, L-ascorbic acid 0.4) at 32 °C for an hour and maintained at room temperature until required. Microelectrodes (75–125 μm exposed fiber) were prepared with T650 fibers (7 μm diameter, Goodfellow), inserted, and sealed into a glass pipette. The carbon-fiber electrode was held at − 0.4 V, and the potential was ramped to + 1.3 V and back to − 0.4 V at 400 V/s every 100 ms. DA release was evoked by a rectangular, electrical increasing input/output (I/O) stimuli (100, 200, 400, and 600 µA, 1.2 ms, monophasic) generated by a DS3 Constant Current Stimulator (Digitimer, Hertfordshire, UK). I/O stimulation was applied every 3 min by a bipolar electrode placed on the surface of the slice over the corpus callosum (CC) to stimulate the local striatal network through the activation of the corticostriatal pathway. The recording electrode was positioned 75 μm into the slice, 100 μm from the center of the tips of the bipolar stimulating electrode, creating an equilateral triangle, within the dorsal striatum. For recordings, brain slices were kept at 32 °C in a chamber perfused at a rate of 1.5 ml/min. To track DA release and data collection, DEMON software^49^ was used. Ten cyclic voltammograms of charging currents were recorded as a baseline before stimulation, and the average was subtracted from data collected during and after stimulation. DA peak amplitude responses were obtained from four to five brains in both DMS and DLS within the rostral-caudal striatum. I/O curves were constructed by plotting the average of DA release peaks at the intensity stimulus of 100, 200, 400, and 600 µA. As an index of DA uptake, the time constants (τ) were obtained from the slope of the DA release transient after the peak reaches its higher amplitude. Also, the ratio τDLS/τDMS from the time constant (τ) was calculated within the same animals and compared between Tg and WT animals. Data represent the average of 4 to 5 mice per genotype (WT and Tg), and for each mouse, 5 to 6 repetitions for each pair of DLS-DMS regions were recorded within the same coronal slice.

#### Dopamine uptake blockage

To assess dopamine transporter functionality, FSCV was carried out in slices incubated with a continuous flow of oxygenated α-CSF containing cocaine hydrochloride (Coc, Sigma-Aldrich. St. Louis, MO, USA, C5776) at a 3 µM concentration^49^. Firstly, DA release was evoked every 3 min for 6 min to assess the basal release amplitude (400 mA stimulus, 1.2 ms, monophasic). Then, the chamber was filled with α-CSF+3 µM COC and DA release was monitored for 15 min. Changes in DA uptake were analyzed by the τ constant as previously described. Data represent the average of 4 to 5 mice per genotype (2 repetitions in each striatal area). For these recordings, a single hemisphere section was used only for one area (DLS or DMS) since exposure to Coc would compromise the integrity of the DArgic terminal function.

### Immunofluorescence and data analysis

Mice were deeply anesthetized with ketamine/xylazine and transcardially perfused with 0.1 M phosphate-buffered saline (PBS) followed by 4% paraformaldehyde buffer. 50 μm-coronal sections were obtained using a vibratome (VT 1000 S. Leica, Wetzlar, Germany). Each immunofluorescence staining was performed in free-floating sections containing rostral, medial, and caudal striatal regions from paired Tg-WT mice. Upon washing, sections were rinsed in a pre-blocking PBS 0.1 M with 0.2% Triton X-100 and 5% normal goat serum (NGS) solution overnight at 4°C. Then, either overnight or 48 h in PBS with 0.2% Triton X-100 and 3% NGS containing a rabbit polyclonal DAT antibody (1/500, AB1591P, Merck KGaA, Darmstadt, Germany). After washing, sections were incubated with a 1/1000 diluted secondary antibody (Alexa 488 anti-rabbit, A32731, Invitrogen, Thermo Fisher Scientific, Waltham, MA USA) for 1 h at room temperature. For long-term preservation, slices were mounted in a fluorescence mounting medium (Agilent, DAKO). Sections were taken through the striatum at three different levels along the rostrocaudal axis, i.e., rostral (corresponding to approximately 1.18 mm in the mouse brain atlas of Paxinos and Franklin (2001), intermediate (approximately 0.02 mm), and caudal (approximately − 1.58 mm). The intermediate striatum was further subdivided into four subregions: dorsolateral (DLS), dorsomedial (DMS), and two ventral areas that were not considered for this study (Gernert et al.^50^). For imaging of the SNc and VTA, coronal slices from equal AP positions (approximately − 3.04, − 3.16, and 3.24 mm from the bregma) were stained as above, using mouse monoclonal TH antibody (1/500, MAB318, Merck KGaA, Darmstadt, Germany), and the following secondary antibody; 1/1000 diluted secondary antibody (Alexa 488 mouse, A32723, Invitrogen, Thermo Fisher Scientific, Waltham, MA USA) for 1 h at room temperature.

Brain sections were imaged using a confocal microscope (Zeiss. Model: LSM 780 Axio inverse, Oberkochen, Germany) at 40 × /1.4NA oil immersion objective (Zeiss) in a rostrocaudal striatal series of at least 3 coronal sections. Two 15-images stacked samples (212.55 × 212.55 µm) were taken for each region (DMS and DLS), and hemisphere. Analysis of Area, Cumulative Frequency distribution (CFD) and Optical Density (OD) were carried out using the free access program Image J (https://imagej.nih.gov/ij/). Next, a maximum intensity z-projection was applied to each stack and a threshold-limiting mask was pre-established to define the specific signal of the primary antibodies. For CFD, images were additionally scanned by the particle analyzer tool (particles smaller than 1.5 µm^2^ were not included in the data acquisition). The bin width was set at 0.1. Data represent averages of CFD of 4 mice in each genotype group (WT and Tg) and 3 repetitions within the mentioned rostrocaudal striatal axis, for each striatal subregion (DLS and DMS). To avoid background signals for the OD analysis, unspecific staining was deducted from immunonegative regions. For SNc and VTA cell counting, 3 images (212.5 × 212.5 µm) were at least acquired per hemisphere of each structure. Non-overlapping regions were imaged with a thin optical Section (2.2 µm). To avoid miscounting, the number of cells was normalized to the area imaged; for SNc the boundary of the structure in the field of view was defined by demarcating the TH fiber tracts. Data reported are the average cell densities of 3 mice in each genotype group, 12 months of age.

### Rotarod task

For testing procedural learning skills, latency to fall was assessed in 3- and 6-month-old Tg and WT littermates on the rotarod task (5 to 6 mice per genotype). The experiment consisted of a habituation phase, where mice acclimated to the apparatus, and a subsequent four-day testing phase, where latency to fall was measured. During the testing day, mice were kept in the room for at least 1 h. On the first day, animals were gently placed on the rod (not rotating) and returned to their enclosures after five minutes approximately. Testing days consisted of 10 trials separated by 3–5 min from each other. The rotating rod (TSE Systems GmbH) speed was set to accelerate from 0 to 40 rpm in 60 s. Recordings were automatically off once mice fell from the rod. Latency to fall was calculated as the average within each day. Mice were weighed at the beginning of the first testing day.

### Statistical analysis

Statistical analysis was carried out using GraphPad Prism 8.4.2. (GraphPad Software, Inc., La Jolla, CA USA). Depending on whether the data followed a normal distribution or not, parametric (Student´s t-test and two-way ANOVA) or non-parametric (unpaired two-tailed Student’s t-test and two-way ANOVA followed by Bonferroni post hoc test) tests were used. For DA release data collected from FSCV, two-way ANOVA was performed to compare the amount of DA release evoked by the different stimuli intensity in the same region (DMS or DLS) of both genotypes. Values for DA uptake were determined by normalizing the time constant from DLS with DMS of the same slice, and then, τ DLS/τ DMS ratios were calculated to an unpaired two-tailed Student´s t-test. Two-way ANOVA was utilized to analyze DA uptake values from DAT blockade experiments, with genotype and time as main factors. Latency to fall data for procedural motor learning was analyzed by using Two-way ANOVA, followed by Bonferroni’s post hoc tests. For the data collected from the immunolabelling, CFD analysis was calculated and averaged per animal and group and Optical Density (OD) level was determined using two-way ANOVA with genotype and regions as main factors. Probabilities of *p* < 0.05, *p* < 0.01, *p* < 0.001 and *p* < 0.0001 were considered significant.

### Supplementary Information

Supplementary Information.

## Data Availability

Data or any information required to re-evaluate or analyze the information exposed in the paper is available upon request to the corresponding author.
