# Supplementary material for: Striatal dopamine neurotransmission is altered in age- and region-specific manner in a Parkinson’s disease transgenic mouse
Source: Sci Rep. 2024 Jan 2;14:164. doi: 10.1038/s41598-023-49600-5 (PMC10761704; doi:10.1038/s41598-023-49600-5)
Supplement: Supplementary file 1 — Supplementary Information. [file 41598_2023_49600_MOESM1_ESM.pdf]

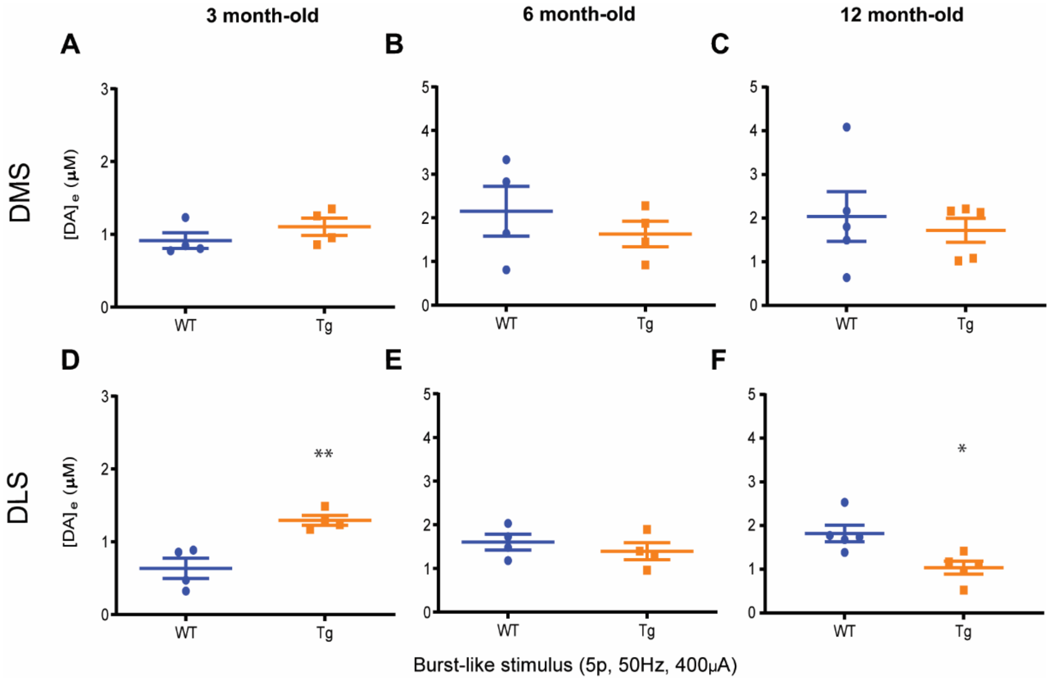

Supplementary Figure 1. Burst-firing stimulus revealed DLS DA release changes between WT and Tg mice.

Evoked DA release induced by a burst-like stimulus in the DMS and DLS of WT and Tg mice at the age of 3 (**A**-**D**), 6 (**B**-**E**), and 12 (**C**-**F**) months. Data represent the mean ± SEM of observed DA release when electrically elicited by a burst-like stimulus (5p, 50Hz, 400 mA). Differences were analyzed carrying out by unpaired two-tailed Student's test at 3 (DMS: $p = 0.2766$; DLS: $t_{(6)} = 4.240$, ** $p < 0.0054$), 6 (DMS, $p = 0.5417$ and DLS, $p = 0.2932$) and 12 (DMS: $p > 0.05$; DLS: $t_{(8)} = 3{,}259$,* $p < 0.05$).

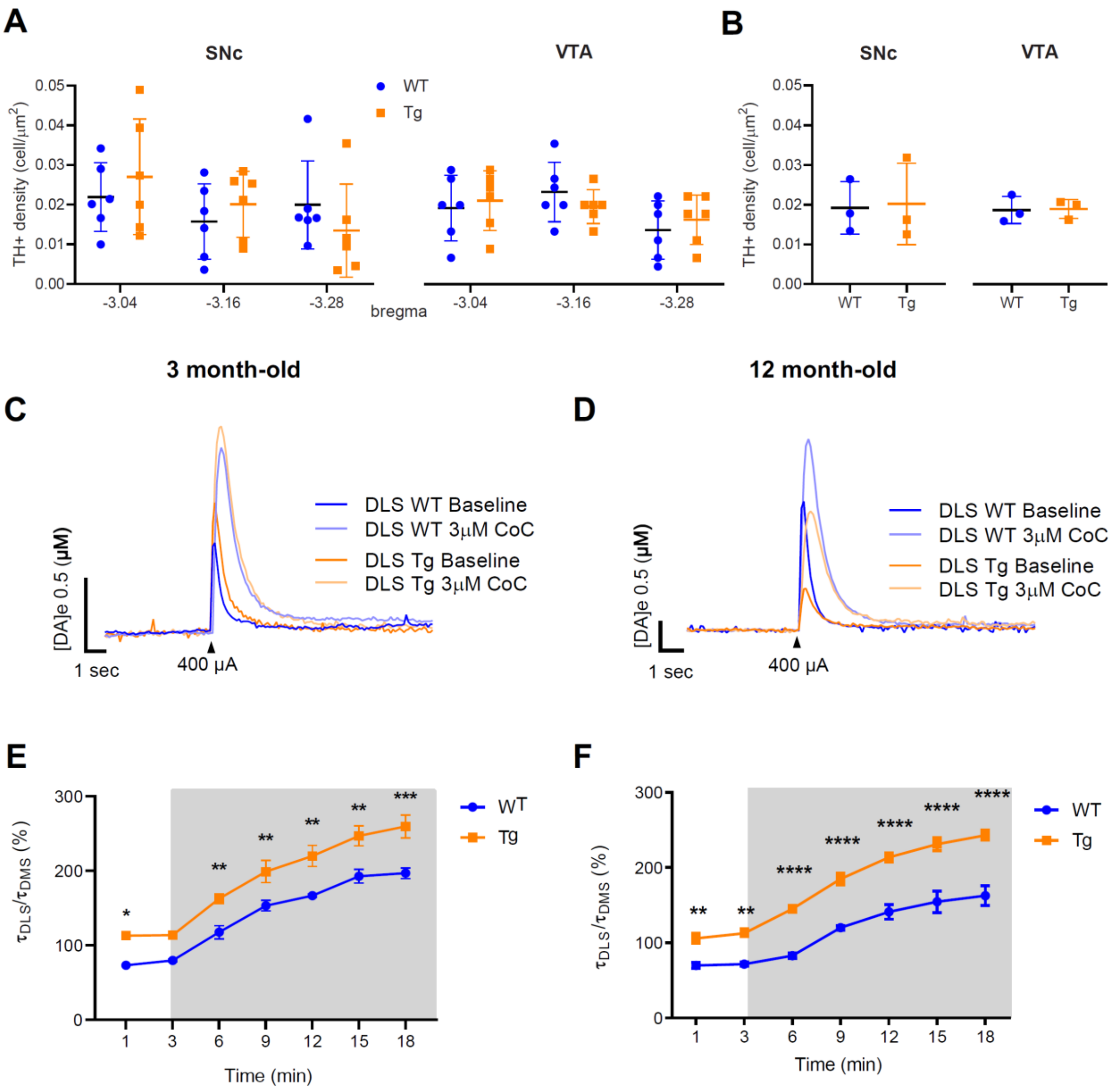

Supplementary Figure 2. Unaltered TH+ cell density in midbrain with concomitant changes in DAT blockade of DA uptake in the DLS of both 3- and 12-month-old Tg mice.

A-B. Quantification of TH+ cell density in the SNc and VTA of 12 month old mice (Wt, blue; Tg, orange). Representative traces for the DLS of WT and Tg mice at the age of 3 (**C**) and 12 months (**D**). Time constant DLS/DMS ratio before and after 15 min exposure to cocaine (Coc, 3 µM, grey blocks) in WT and Tg at 3- (**E**), and 12-month-old (**F**) mice. (Bonferroni's post hoc tests: * $p<0.05$, ** $p<0.01$, *** $p<0.001$, **** $p<0.0001$). Data represent mean ± SEM from two repetitions of 4 to 5 mice.

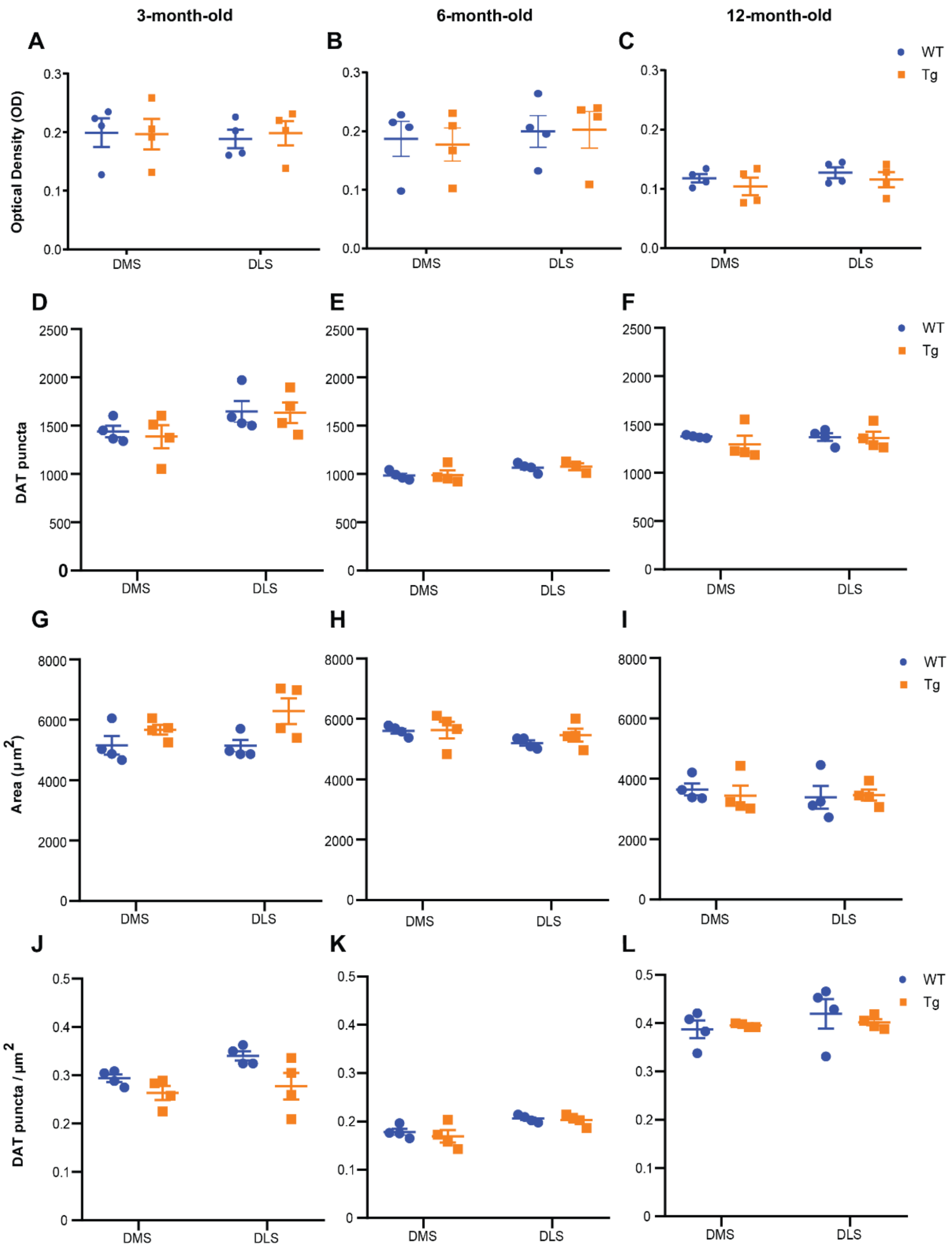

Supplementary Figure 3. Overexpression of h-α-syn did not change either the relative protein distribution of DAT or DAT-like puncta density. Graphs represent the mean ± SEM of the optical density (OD; A-C) as well as DAT puncta (D-F), area (G-I), and DAT puncta/area (J-L) values in the DMS and DLS of WT (blue) and Tg (orange) animals at 3, 6, and 12 months of age. Two-way ANOVA was used for statistical analysis, revealing no significance in comparing different regions between and within groups ($p > 0.05$).
